# Supplementary material for: Modeling glioblastoma heterogeneity as a dynamic network of cell states
Source: Mol Syst Biol. 2021 Sep 16;17(9):e10105. doi: 10.15252/msb.202010105 (PMC8444284; doi:10.15252/msb.202010105)
Supplement: Supplementary file 6 — Source Data for Figure 5 [file MSB-17-e10105-s004.zip › Figure5A_sourcedata/GSEA_3017/nosigdb_stateD.GseaPreranked.1622795010397/FELDMAN_2020_G1.html]

Details for gene set FELDMAN\_2020\_G1[GSEA]

|  || Dataset | state23017 |
| Phenotype | NoPhenotypeAvailable |
| Upregulated in class | na\_neg |
| GeneSet | FELDMAN\_2020\_G1 |
| Enrichment Score (ES) | -0.65447396 |
| Normalized Enrichment Score (NES) | -3.5109174 |
| Nominal p-value | 0.0 |
| FDR q-value | 0.0 |
| FWER p-Value | 0.0 |
Table: GSEA Results Summary

  

Fig 1: Enrichment plot: FELDMAN\_2020\_G1      
 Profile of the Running ES Score & Positions of GeneSet Members on the Rank Ordered List

  

| PROBE | GENE SYMBOL | GENE\_TITLE | RANK IN GENE LIST | RANK METRIC SCORE | RUNNING ES | CORE ENRICHMENT || 1 | PTTG1 |  |  | 14 | 0.490 | -0.0025 | No |
| 2 | HMGB3 |  |  | 80 | 0.311 | -0.0497 | No |
| 3 | TROAP |  |  | 109 | 0.283 | -0.0673 | No |
| 4 | LSM5 |  |  | 123 | 0.274 | -0.0729 | No |
| 5 | BIRC5 |  |  | 130 | 0.271 | -0.0729 | No |
| 6 | MKI67 |  |  | 206 | -0.254 | -0.1291 | No |
| 7 | EXOSC8 |  |  | 347 | -0.268 | -0.2379 | No |
| 8 | CYR61 |  |  | 555 | -0.292 | -0.4005 | No |
| 9 | SIVA1 |  |  | 567 | -0.293 | -0.4041 | No |
| 10 | PRC1 |  |  | 645 | -0.310 | -0.4610 | No |
| 11 | DNMT1 |  |  | 659 | -0.312 | -0.4660 | No |
| 12 | RAD21 |  |  | 674 | -0.314 | -0.4717 | No |
| 13 | TMPO |  |  | 688 | -0.317 | -0.4765 | No |
| 14 | KNSTRN |  |  | 690 | -0.317 | -0.4716 | No |
| 15 | S100B |  |  | 705 | -0.320 | -0.4772 | No |
| 16 | KIF4A |  |  | 706 | -0.320 | -0.4714 | No |
| 17 | CDC20 |  |  | 793 | -0.341 | -0.5350 | No |
| 18 | H2AFX |  |  | 810 | -0.347 | -0.5418 | No |
| 19 | CENPM |  |  | 818 | -0.349 | -0.5412 | No |
| 20 | MAD2L1 |  |  | 819 | -0.350 | -0.5349 | No |
| 21 | BUB3 |  |  | 826 | -0.351 | -0.5334 | No |
| 22 | CKAP2 |  |  | 900 | -0.375 | -0.5859 | No |
| 23 | TK1 |  |  | 902 | -0.375 | -0.5800 | No |
| 24 | TUBB4B |  |  | 916 | -0.380 | -0.5837 | No |
| 25 | USP1 |  |  | 930 | -0.386 | -0.5873 | No |
| 26 | HAT1 |  |  | 933 | -0.387 | -0.5819 | No |
| 27 | PSIP1 |  |  | 934 | -0.388 | -0.5749 | No |
| 28 | CCDC34 |  |  | 1013 | -0.421 | -0.6306 | No |
| 29 | CMC2 |  |  | 1017 | -0.425 | -0.6254 | No |
| 30 | MIS18A |  |  | 1020 | -0.427 | -0.6194 | No |
| 31 | TPX2 |  |  | 1034 | -0.437 | -0.6221 | No |
| 32 | KIF22 |  |  | 1051 | -0.446 | -0.6270 | No |
| 33 | SMC2 |  |  | 1062 | -0.452 | -0.6270 | No |
| 34 | DEPDC1 |  |  | 1074 | -0.457 | -0.6277 | No |
| 35 | PLK1 |  |  | 1082 | -0.460 | -0.6251 | No |
| 36 | ZWINT |  |  | 1099 | -0.467 | -0.6297 | No |
| 37 | DLG1 |  |  | 1100 | -0.469 | -0.6212 | No |
| 38 | PCNA |  |  | 1142 | -0.504 | -0.6454 | Yes |
| 39 | VRK1 |  |  | 1143 | -0.505 | -0.6363 | Yes |
| 40 | CKAP2L |  |  | 1147 | -0.508 | -0.6296 | Yes |
| 41 | TMEM106C |  |  | 1148 | -0.509 | -0.6205 | Yes |
| 42 | NUSAP1 |  |  | 1154 | -0.515 | -0.6153 | Yes |
| 43 | GMNN |  |  | 1155 | -0.516 | -0.6060 | Yes |
| 44 | DBF4 |  |  | 1157 | -0.520 | -0.5974 | Yes |
| 45 | RRM1 |  |  | 1166 | -0.534 | -0.5943 | Yes |
| 46 | ORC6 |  |  | 1179 | -0.551 | -0.5941 | Yes |
| 47 | CENPU |  |  | 1182 | -0.555 | -0.5858 | Yes |
| 48 | CDCA3 |  |  | 1187 | -0.563 | -0.5789 | Yes |
| 49 | ASPM |  |  | 1189 | -0.567 | -0.5695 | Yes |
| 50 | FEN1 |  |  | 1191 | -0.569 | -0.5601 | Yes |
| 51 | NUF2 |  |  | 1194 | -0.574 | -0.5514 | Yes |
| 52 | DHFR |  |  | 1197 | -0.576 | -0.5426 | Yes |
| 53 | CKS1B |  |  | 1198 | -0.577 | -0.5323 | Yes |
| 54 | GTSE1 |  |  | 1210 | -0.602 | -0.5303 | Yes |
| 55 | CLSPN |  |  | 1212 | -0.605 | -0.5203 | Yes |
| 56 | CENPK |  |  | 1220 | -0.619 | -0.5148 | Yes |
| 57 | DUT |  |  | 1223 | -0.633 | -0.5051 | Yes |
| 58 | SMC4 |  |  | 1224 | -0.638 | -0.4936 | Yes |
| 59 | CKS2 |  |  | 1226 | -0.641 | -0.4828 | Yes |
| 60 | ANLN |  |  | 1229 | -0.646 | -0.4728 | Yes |
| 61 | CTGF |  |  | 1234 | -0.654 | -0.4643 | Yes |
| 62 | CENPA |  |  | 1243 | -0.679 | -0.4586 | Yes |
| 63 | AURKB |  |  | 1250 | -0.700 | -0.4509 | Yes |
| 64 | CCNA2 |  |  | 1255 | -0.716 | -0.4412 | Yes |
| 65 | UBE2T |  |  | 1256 | -0.719 | -0.4283 | Yes |
| 66 | CDCA2 |  |  | 1258 | -0.727 | -0.4160 | Yes |
| 67 | HMGB2 |  |  | 1263 | -0.736 | -0.4060 | Yes |
| 68 | KIF23 |  |  | 1265 | -0.741 | -0.3935 | Yes |
| 69 | AURKA |  |  | 1271 | -0.763 | -0.3838 | Yes |
| 70 | TYMS |  |  | 1272 | -0.770 | -0.3700 | Yes |
| 71 | ATAD2 |  |  | 1275 | -0.775 | -0.3576 | Yes |
| 72 | NDC80 |  |  | 1278 | -0.810 | -0.3447 | Yes |
| 73 | ESCO2 |  |  | 1283 | -0.846 | -0.3327 | Yes |
| 74 | CALM2 |  |  | 1286 | -0.878 | -0.3186 | Yes |
| 75 | MALAT1 |  |  | 1289 | -0.884 | -0.3043 | Yes |
| 76 | SPC25 |  |  | 1294 | -0.932 | -0.2907 | Yes |
| 77 | RRM2 |  |  | 1295 | -0.933 | -0.2740 | Yes |
| 78 | KPNA2 |  |  | 1297 | -0.972 | -0.2573 | Yes |
| 79 | FBXO5 |  |  | 1301 | -0.992 | -0.2419 | Yes |
| 80 | NCAPG |  |  | 1303 | -1.015 | -0.2244 | Yes |
| 81 | IGFBP5 |  |  | 1307 | -1.052 | -0.2079 | Yes |
| 82 | HIST1H1E |  |  | 1309 | -1.319 | -0.1850 | Yes |
| 83 | HIST1H1C |  |  | 1310 | -1.391 | -0.1600 | Yes |
| 84 | CDK1 |  |  | 1311 | -1.433 | -0.1342 | Yes |
| 85 | UBE2C |  |  | 1312 | -1.466 | -0.1078 | Yes |
| 86 | TOP2A |  |  | 1313 | -1.528 | -0.0803 | Yes |
| 87 | HIST1H1B |  |  | 1318 | -1.922 | -0.0490 | Yes |
| 88 | HIST1H4C |  |  | 1320 | -2.769 | 0.0000 | Yes |
Table: GSEA details [plain text format]

  

Fig 2: FELDMAN\_2020\_G1: Random ES distribution      
 Gene set null distribution of ES for **FELDMAN\_2020\_G1**
